# Isolated Deep Infiltrating Endometriosis of the Sciatic Nerve: A Case Report and Overview of the Literature

**DOI:** 10.3390/medicina59122161

**Published:** 2023-12-13

**Authors:** Milena Zamurovic, Ana Tomic, Katarina Djordjevic, Sara Simanic, Jelena Sopta, Lukas Rasulic, Ljubica Simic, Jovan Jevtic, Olga Nedeljkovic-Arsenovic, Marija Rovcanin

**Affiliations:** 1 Clinic for Gynecology and Obstetrics, Narodni Front, Kraljice Natalije 62, 11000 Belgrade, Serbia; 2 Faculty of Medicine, University of Belgrade, Dr Subotica Starijeg 8, 11000 Belgrade, Serbia; 3 University Clinical Center of Serbia, Center for Radiology and Magnetic Resonance Imaging, 11000 Belgrade, Serbia; 4 Department for Pathology, Faculty of Medicine, University of Belgrade, Dr Subotica 4/2, 11000 Belgrade, Serbia; 5 Department of Peripheral Nerve Surgery, Functional Neurosurgery and Pain Management Surgery, Clinic for Neurosurgery, Clinical Center of Serbia, Faculty of Medicine, University of Belgrade, Dr Koste Todorovica 4, 11000 Belgrade, Serbia

**Keywords:** female healthcare, endometriosis, sciatic nerve, diagnosis, pharmacotherapy, outcome

## Abstract

Isolated deep infiltrating endometriosis (DIE) of sacral nerve roots or major pelvic nerves, including the sciatic nerve, is considered to be extremely rare. Due to the overlap with sciatica symptoms, the diagnosis of sciatica DIE is difficult yet crucial, as it results in permanent neural damage if left untreated. We report a case of a 45-year-old woman who experienced a three-year-long and recently exacerbating pain in her right leg, accompanied by a tingling sensation and weakness in her right leg and foot, with difficulty walking. In between regular menstrual bleedings, when her aforementioned symptoms worsened, she had been experiencing mild 10-day extra-cyclical bleeding. Her neurologist’s, orthopedist’s, and gynecological examinations were unremarkable. Magnetic resonance imaging (MRI) showed an infiltrative lesion on the right sciatic nerve that was immunohistochemically confirmed to be endometriosis. The patient was treated with gonadotropin-releasing hormone analogues (GnRHa), which led to a significantly diminished size of the lesion on the control MRI, and endometriosis remission was obtained. For persistent mild, but cyclical, pain and muscle weakness, continuous progestagnes were administered, with advice for physical therapy provided for her neuro-muscle rehabilitation and a scheduled check-up in 6 months.

## 1. Background

Endometriosis is a chronic, inflammatory, estrogen-dependent disease resulting from the presence of endometrial-like tissue outside the uterus [1]. This condition affects approximately 6–10% of women in their reproductive years [2], but in certain studies, the prevalence of clinically suspected cases has reached 11.4% [3] or even 49–75% in adolescents with chronic pelvic pain [1]. This disease is regarded as a significant public health issue that greatly impacts the well-being of women. It is a major cause of infertility and imposes a considerable economic burden [4].

The depiction of endometriosis as a disorder resulting only in symptoms associated with cyclical changes in the endometrial-like tissue is archaic and no longer accurately represents the whole extent and manifestations of the condition. The clinical appearance of the disease is diverse, with a wide range of pelvic lesions, and there is limited comprehension of how the disease affects locations outside of the female genital tract, hence the reason endometriosis is recognized as a systemic disease [5]. Although the disease can be heterogeneous, three major phenotypes are recognized: superficial peritoneal, ovarian endometriosis, and deep infiltrating endometriosis (DIE) [4]. Even though the DIE prevalence is considered to be around 17%, the DIE of sacral nerve roots or major pelvic nerves is considered a rarity that occurs in less than 0.1% [6]. Although DIE solely affecting the nerves without any observable endometriosis within the pelvic cavity is considered to be rather uncommon, Possover et al. documented isolated endometriosis of the sciatic nerve in 27 patients over a five-year period [7]. Possover’s [8] 17-year-long follow-up study showed 267 cases of endometriotic lesions with no additional manifestations within the peritoneal cavity and no DIE of the parametrium. Endometriotic lesions impacted solely the sciatic nerve in approximately 67 cases, with no influence on any other pelvic tissue, such as surrounding muscles (4 cases per year, on average).

Due to the overlap with sciatica symptoms, Salazar-Grueso and Roos [9] reported a mean interval of around 4 years between the onset of symptoms and diagnosis. This disorder should be taken into account when making a differential diagnosis in young women who are of reproductive age, particularly if they have a history of dysmenorrhea, demonstrating a possible association with symptoms of sciatica [10].

Diagnosis is difficult yet crucial, as DIE results in permanent neural damage if left untreated [11]. This cyclic tissue injury and repair, combined with stimulation by Tumor Growth Factor-β1 secreted from activated platelets and other immune cells, as well as neuropeptides secreted from sensory nerve fibers, result in epithelial-mesenchymal transition (EMT) and fibroblast-to-myofibroblast transdifferentiation (FMT). EMT and FMT lead to increased cellular contractility and collagen production, ultimately causing fibrosis and permanent nerve damage [12]. Endometrial and endometriotic epithelial cells also contain various cancer mutations, which may be associated with the formation of pelvic endometriosis or ovarian cancer [13]. Although exceedingly uncommon, instances of carcinoma originating from a localized area of sciatic endometriosis have been documented, exhibiting no notably distinct clinical symptoms compared to noncancerous endometriosis lesions [14], further emphasizing the importance of prompt diagnosis.

This article presents a case of isolated DIE of the sciatic nerve and discusses the complex issues behind the pathogenesis, diagnosis, and treatment of this type of endometriosis.

## 2. Case Presentation

**Figure 1:**
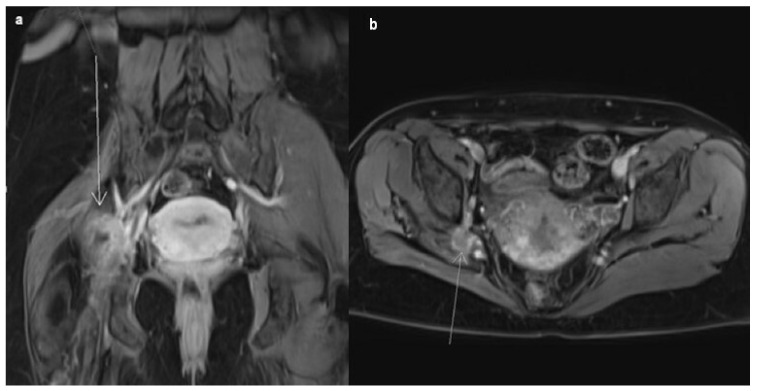
Postcontrast coronal (**a**) and axial (**b**) T1w FS MRI images present a heterogeneous mass in the right obturator fossa (white arrows), vaguely demarcated from the neurovascular pedicle. Note the large fibroids in the anterior uterine wall (picture (**b**)).

A 45-year-old female patient came to see a general practitioner because of a three-year-long mild and now exacerbating pain in her right leg, accompanied by a tingling sensation. She experienced weakness in her right leg and even more so in her right foot, with difficulty walking, which has now disrupted her daily life. Occasionally, in between regular menstrual bleedings that occurred every 28 days, she had been experiencing mild, 10-day-long extra-cyclical bleeding. Based on the presented symptoms, the patient was further examined by a neurologist, orthopedist, gynecologist, and neurosurgeon. The pain was first localized in the right gluteal region and then further spread through the posterior compartment of the whole leg. As mentioned, the pain was accompanied by upper-thigh tingling that also progressed downward to the foot. Assessing muscle strength during neurological examination, muscles on the right lower limb were found to be discretely weaker but able to contract and provide resistance. Moreover, when maximum resistance was exerted, the muscles were unable to maintain the contraction (graded 4+/5 muscle strength). The orthopedist’s examination yielded no noteworthy findings. The only gynecological symptom reported by the patient was intermenstrual bleeding. In terms of extra-cyclical bleeding, all diagnostic procedures were carried out in order to find the possible organic cause. Examinations of the patient’s hormonal status were also carried out. All findings were within physiological limits and ruled out the existence of organic and hormonal causes of intermenstrual bleeding, i.e., the existence of endometrial polyps, cervical polyps, submucosal myomas, and colposcopic lesions that can bleed, but also hormonal-cause and abnormal uterine bleeding in the middle of the cycle. Furthermore, the gynecologist reported normal results from the Pap smear and colposcopy. Transvaginal color Doppler ultrasound showed a uterus in a marked retroverted (RVF) position with inhomogeneous and irregular contours; showed multiple non-cavity-distorting myomas in its anterior wall, up to 5 cm in size; and ruled out the existence of lesions on the genital organs that could raise suspicion of pelvic endometriosis. The pain sensitivity of the tissue of the uterus, parametrium, and adnexal region was not determined by pelvic examination. All tumor markers (cancer antigen 125, carcinoembryonic antigen, cancer antigen 19.9), as well as inflammation markers (c-reactive protein, erythrocyte sedimentation rate), were within reference values. Therefore, magnetic resonance imaging (MRI) of the small pelvis was advised. Examination was performed using a 1.5-T scanner (Avanto fit, Siemens Medical Systems, Erlangen, Germany) with a master gradient system (45 mT/mpeak gradient amplitude, 200 m/T/s slew rate) and an eighteen-element array body surface coil and thirty-two element spine coil. The bladder with perivesical adipose tissue, the lineages of both ovaries, the parametrium with ischiorectal fossae, as well as the visible digestive tract and mesorectal and pararectal adipose tissue, were morphologically preserved, without any hiperintense solid or cystic endometriosis-like mass lesions. The body of the uterus was clearly demarcated by several intramural fibroids, with the largest one being the right anterior wall, measuring approximately 5 cm. None of the identified alterations has the potential to elucidate the neurological or gynecological symptoms observed in our patient. A minimal amount of free fluid was visible in the pelvis. It could be seen that the right gluteal group of muscles was atrophic, as in the right external obturator muscle compartment, and a vaguely demarcated heterogeneous formation was registered, transversely measuring approximately 30 mm and heterogeneously increasing the signal intensity post contrast. The whole formation was vaguely demarcated from the neurovascular pedicle, hence preventing the exclusion of the potential presence of an infiltrative lesion with an unknown cause (Figure 1).

Given the unclear etiology and highly possible infiltration of the neurovascular pedicle in the obturator fossa, the patient was referred to a neurosurgeon, who performed an explorative biopsy. Using the right transgluteal approach, the piriform muscle and piriform foramen were accessed, where the diffusely altered sciatic nerve was observed. The sciatic nerve was compressed by calcified fibrous tissue in the vicinity of the piriform foramen. With the decompression and nerve deliberation, accessible parts of the nerve were microscopically inspected. Sciatic nerve tissue was diffusely altered and thickened, permeated with cysts filled with dark liquid. As the lesions’ etiology had not been readily apparent with suspicion of a malignant process and nerve infiltration, samples of the altered nerve tissue were removed and sent for a histopathological analysis. Postoperatively, a newly developed right foot drop was observed, so the patient was discharged with advice for physical therapy and rehabilitation.

**Figure 2:**
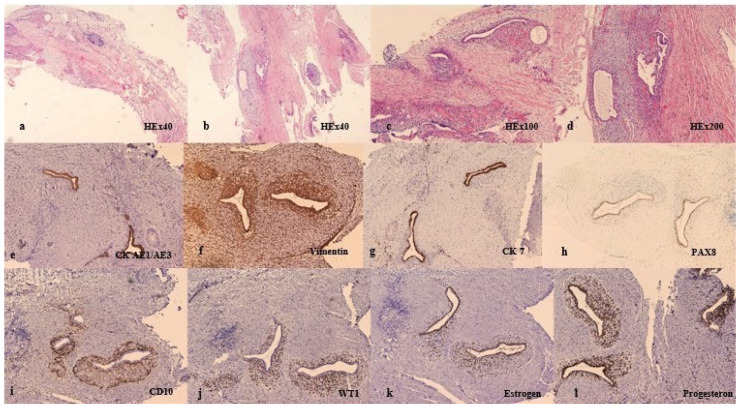
Pathology analyses: (**a**–**d**): neural and perineural tissue infiltrated by endometrial types of glands and stroma; HEx40 (Mayer’s hematoxylin 05-06002/L, Bio Optica, Milano, Italy; Eosin Y 1% aqueous solution 05-10007/L, Bio Optica, Milano, Italy) (1–2) and HEx100 (3–4); (**e**–**l**): immunohistochemical features of the lesion: epithelial cells showed diffuse and intensive cytoplasmic CK AE1/AE3, CK7, and EMA positivity, as well as PAX8, estrogen, and progesterone nuclear positivity. On immunohistochemical analyses, stromal cells had membranous CD10 together with high nuclear WT1, estrogen, and progesterone positivity.

Immunohistochemically, the glands were positive for CK AE1/AE3 (DAKO, Agilent, Santa Clara, CA, USA, Monoclonal Mouse Anti-Human Cytokeratin Clones AE1/AE3-M3515, dilution 1:100, pH6), CK7 (DAKO, Agilent, Santa Clara, CA, USA, Monoclonal Mouse Anti-Human Cytokeratin 7, Clone OV-TL 12/30/M7018, dilution 1:100, pH9), and PAX8 (Cell Marque, Rocklin, CA, USA, Mouse Monoclonal Primary Antibody PAX8, Clone MRQ-50/06523927001, Ready-to-Use, pH8); stromal cells were positive for CD10 (DAKO, Agilent, Santa Clara, CA, USA, Monoclonal Mouse Anti-Human CD 10, Clone 56C6/IR648, Ready-to-Use, pH 9), WT1 (DAKO, Agilent, Santa Clara, CA, USA, Monoclonal Mouse Anti-Human Wilms Tumor 1 Protein, Clone 6F-H2/IR055, Ready-to-Use, pH9), and Vimentin (Novocastra, Leica Biosystems Newcastle, Newcastle, UK, Mouse Monoclonal Antibody Vimentin, Clone V9/NCL-L-VIM-V9, dilution 1:200, pH9); while estrogen (Novocastra, Leica Biosystems Newcastle, UK, Mouse Monoclonal Antibody Estrogen Receptor, Clone 6F11/NCL-L-ER-6F11, dilution 1:50, pH6) and progesterone receptors (Novocastra, Leica Biosystems Newcastle, UK, Mouse Monoclonal Antibody Progesterone Receptor, Clone 16/NCL-L-PGR-312, dilution 1:200, pH9) were positive in both stromal cells and glands. Based on the morphological and immunohistochemical characteristics, a diagnosis of endometriosis was made (Figure 2).

**Figure 3:**
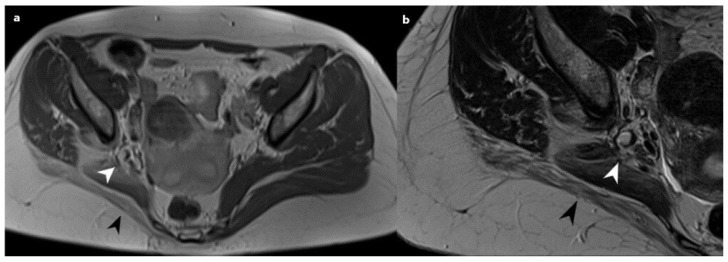
Post-GnRHa treatment; an axial T1 (**a**) and T2 (**b**) weighted MRI scan showing a now reduced-sized right sciatic endometriosis nodule (white arrowheads); note gluteal muscle atrophy (black arrowheads).

Based on the histological observations indicating the presence of endometriosis in the sciatic nerve, the patient was subsequently sent to a gynecologist for a follow-up examination and the prescription of appropriate treatment. The patient was recommended to initiate treatment with GnRH analogs (Triptorelin 3.75 mg) for 6 months (6 cycles) and then to return for a repeat examination. On the 6-month follow-up, the patient confirmed that she tolerated the treatment with GnRH analogs well. After the completion of GnRHa treatment, a follow-up hormonal status and MRI were performed. Scans revealed alterations in the right sciatic nerve, characterized by increased thickness in the proximal segments, with subsequent piriform muscle fatty atrophy. The right ischiatic nerve was observed to be attached to a now reduced-sized endometriosis nodule, visible as a hyperintense zone measuring 7 mm. (Figure 3). Right gluteal muscle group atrophy persisted. In addition to the observed alterations in the DIE lesion and the sciatic nerve, no significant differences were observed when comparing the pre-biopsy MRI scans.

The consequent absence of heavy cyclical sciatica with medically induced amenorrhea and the notable regression of the endometriosis lesion on the latest MRI scans led to the conclusion that the patient had achieved a state of remission. Regarding sciatica symptoms, the patient saw notable improvements in subjective well-being. There was no occurrence of intermenstrual bleeding, and the intensity of cyclical pains was greatly reduced. In the rare instances when pain did arise, they were tolerable, resulting in the seldom use of pain relievers. The patient highlights a substantial improvement in the quality of her everyday life and her successful return to work. As cyclical mild pain in her right leg persisted, in order to improve symptom management and mitigate the progression of the condition, a continuous therapy regimen with oral progestagen (Norethisterone, 5 mg twice daily) was advised for the patient, taking into account her age and normal hormonal status. For the remaining right-leg muscle weakness as well as the MRI-noted atrophy of the right gluteal and piriform muscles, the patient was to continue previously advised physical therapy and rehabilitation. The patient is still being followed and was scheduled for a 6-month check-up.

## 3. Discussion

The presented case is an uncommon form of endometriosis, with the disease affecting only the right sciatic nerve and no visible endometrial nodules in the pelvic peritoneum or other parts of the pelvic cavity.

The difficulty associated with this particular form of solitary endometriosis comes from elucidating how it develops. The notion of proposing implantation and retrograde menstruation as the main mechanisms behind this condition is scientifically refuted. The same stands for the hypotheses of coelomic metaplasia, suggesting that endometriosis arises from the conversion of cells that line the pelvic peritoneum that have the same embryological origin as certain cells that line female reproductive organs, like the ovaries [4]. However, there is no evidence of a common embryological predecessor when it comes to somatic nerves [15]. Recent hypotheses propose that endometrial cells have the ability to travel through the circulation and lymphatic systems, ultimately appearing in the retroperitoneum [16]. Hence, the most probable reason for the occurrence of sciatic endometriosis, as well as its presence in other atypical locations other than the peripheral nerves, is that endometrial cells have the ability to spread through the lymphatic and/or vascular system [17]. Upon being inserted into the peripheral nerve, ectopic endometrial nodules exhibit highly invasive behavior as they penetrate the epineurium and perineurium.

A meta-analysis by Vercellini et al. [18] showed that the majority of patients with sciatic DIE had right-side lesions. The main sciatic DIE manifestation is cyclical pain that resembles typical sciatica. Perineural and intraneural endometriosis cause pain mainly because of nerve damage by cyclical inflammation, with ectopic uterine tissue spreading through the epineurium and perineurium [19]. The local production in the endometrial stroma of prostaglandins, interleukins, and histamine also exacerbates patients’ symptoms. It is apparent that endometriosis implants stimulate the formation of their own neural and vascular branches through neuroangiogenesis, which influences dorsal root neurons within the central nervous system, increasing pain perception in patients [20].

Additionally, physiological hormonal shifts that lead to cyclic menstrual bleeding also affect the ectopic endometrium, leading to intrafascicular hemorrhage [21], as areas of blood collections are commonly seen within the mass on MRI [22]. This leads to further nerve compression [21], as well as aggravation of perineural inflammation [23] and worsening of the sciatic pain. Eventually, cysts filled with dark hemorrhagic content (noted as “chocolate cysts” in the literature) are formed and are usually seen during the laparoscopic exploration of the unknown origin of sciatica [11]. These cysts grow with each menstrual bleeding, causing constant pressure and nerve damage, finally resulting in worsening cyclical sciatica or even constant pain with significant menstrual exacerbations [8].

Lesions that predominantly affect the sciatic nerve without infiltrating surrounding muscle or neurovascular structures are presented as cyclical sciatica in over 75% of cases, less frequently as constant sciatica [8]. They experience worsening sciatic pain that usually begins in the gluteal area and radiates down the posterolateral side of the leg and heel [22]. Consistent with our case and the mass localization, patients more commonly exhibit leg motor weakness and sciatic dermatome hypoesthesia [24]. Interestingly, Possover noted that less than 20% of isolated sciatic endometriosis cases within the first year of experiencing pain are accompanied by neurologic disorders such as foot drop, Trendelenburg gait, sacral hypoesthesia, and gluteal atrophy [8]. Other symptoms that usually occur are related to abdominal-pelvic organ involvement: dysmenorrhea, chronic pelvic pain, right iliac fossa pain, dysuria, dyspareunia, and infertility [25]. Therefore, it is of great importance to consider a detailed history to suspect this rare cause of sciatica. Diagnosis is, thus, challenging, but “cyclical sciatica”, with worsening symptoms during menstrual bleeding, should raise suspicion about endometriosis [11].

Given the significant improvements in diagnostic imaging techniques in the fields of ultrasound and MRI, exploratory laparoscopy should no longer be employed for the purpose of diagnosing endometriotic lesions [4]. As the imaging modality of choice is MRI, on T1W MRI scans, endometriosis lesions reveal a high signal intensity, while T2W scans reveal high and low signal intensities [26]. Not all cases of sciatic endometriosis can be identified on MRI, and, frequently, further investigations are needed to rule out other potential benign and malignant etiologies. Where there is uncertainty regarding the imaging diagnosis, mainly when differentiating sciatic DIE from malignant peripheral nerve sheath tumors, CT-guided biopsy could be the diagnostic modality of choice [14]. Some authors have reported successfully using high-resolution ultrasound to diagnose endometriosis of the pelvic nerve, which could have potential benefits compared to MRI due to its availability and ease of access [27]. Finally, if no imaging modality brings adequate results or is unavailable, an exploration of the lumbosacral space and a histopathological exam offer the exact diagnosis [28].

As surgery is the most often reported treatment of choice for endometriosis of the sciatic nerve, there are limited reports of successful hormonal treatment [29]. Even when applied, this type of treatment has been shown to only ease the pain and other symptoms of endometriosis without eliminating the endometriotic lesion itself [30]. Additionally, lesions around the nerves are often progressive, so prompt surgical intervention is necessary to prevent permanent nerve damage and muscle atrophy [9]. A consideration of endopelvic pathology, such as endometriosis, must be raised in patients with sacral radiculopathy or sciatica of unknown etiology, followed by adequate imaging and a laparoscopic assessment of the sacral plexus and sciatic nerve [7]. Laparoscopic neurolysis is an aetiological procedure that assists in alleviating neurological issues caused by nerve infiltration and compression [31], leading to a significant reduction in pain symptoms [6]. Full resection of the endometriosis lesion and segmental nerve resection with postoperative medical treatment and intensive, at least 3-year-long, physiotherapy give good results in sciatic nerve function recovery. At the 5-year follow-up, Possover et al. reported that all patients exhibited significant pain reduction and recovery of normal gait, including the ability to climb stairs [32], as the overall quality of life of patients substantially improved after surgery [33]. Even though microsurgical resection is considered to be the best treatment for sciatic endometriosis, total recovery is unlikely in advanced cases [34].

Since endometriosis is a chronic condition, it is not uncommon for recurrences to occur, in which case hormonal therapy is indicated. The outcome in terms of pain relief and the recurrence rate is better when the hormonal treatment-free interval is as short as possible [35]. The continuous administration of combined oral contraceptives (COCs) is traditionally regarded as the first-line treatment for dysmenorrhea and chronic pelvic pain in endometriosis, as they are efficacious, safe, well-tolerated, and cost-effective [36]. But, recently, it has been established that a potential adverse effect of the long-term use of COCs is the recurrence and further progression of endometriosis. It appears that these patients may do better with oral progestin-only treatment as first-line therapy because progestins have demonstrated benefits in reducing pain and suppressing the anatomic extent of endometriotic lesions. Unlike COCs, oral progestins alone can be used at any age, and they do not increase the risk of thrombosis [37]. Periodic follow-ups are necessary, as progestins are unsuccessful in a third of symptomatic women [5], mainly caused by progesterone receptor deficiency with subsequent progesterone resistance [13]. Gonadotropin-releasing hormone agonists (GnRHa) are used in patients with exacerbated and persistent symptoms [36]. However, due to their high cost and increased side effects, they are given short-term, with “add-back” hormone treatment given for the prevention of symptom relapse as well as adverse effects of GnRHa [35,36].

Given that current treatments for endometriosis primarily include contraception and require long-term use, resulting in decreased fertility and a notable likelihood of the condition recurring, surgery is the preferred treatment option for young women. There is presently no available medication for improving fertility in women whose inability to conceive is linked to endometriosis [34]. However, in perimenopausal women, continuous oral treatment may be a valuable treatment option if surgery is not possible. A case report of 46- and 50-year-old patients with endometriosis that involved sciatic nerve that were treated only with gonadotropin-releasing hormone agonists reported that medical treatment resulted in complete regression of the lesion with the restoration of normal nerve structure, accompanied by the relief of symptoms [27].

## 4. Conclusions

The diagnosis of isolated sciatic endometriosis could present a challenge and require a multidisciplinary approach, especially when the lesion is solely located in the sciatic nerve. Also, it is important to think of sciatic endometriosis in the case of cyclic sciatica, which could be challenging in deciding on the treatment of pain due to sciatica by neurologists and neurosurgeons. Typical sciatica, with cyclical worsening during menstrual bleeding, should raise suspicion about endometriosis. MRI was the imaging modality of choice for the diagnosis of endometriosis, which was confirmed on histopathology. Medical treatment with GnRHa, followed by continuous progestins, should be considered a treatment of choice for obtaining remission and improving the daily function and quality of life of patients.

## Data Availability

Data sharing is not applicable to this article as no datasets were generated or analyzed during the current study.
